# Peter V. Carroll

**Published:** 1896-06

**Authors:** 


Peter V. Carroll, husband of Dr. Jane W. Carroll and father of Dr. Evangeline Carroll, died at his home, in Buffalo, April 20, 1896, of acute uremic poisoning. He was 48 years of age and had been a sufferer from chronic Bright's disease for a number of years. He was a prominent business man and well beloved on account of his excellent mental and moral qualities. He leaves a wife and ten children.

